# Repeated, long-distance migrations by a philopatric predator targeting highly contrasting ecosystems

**DOI:** 10.1038/srep11202

**Published:** 2015-06-09

**Authors:** James S. E. Lea, Bradley M. Wetherbee, Nuno Queiroz, Neil Burnie, Choy Aming, Lara L. Sousa, Gonzalo R. Mucientes, Nicolas E. Humphries, Guy M. Harvey, David W. Sims, Mahmood S. Shivji

**Affiliations:** 1 The Guy Harvey Research Institute, Nova Southeastern University Oceanographic Center, 8000 North Ocean Drive, Dania Beach, Florida 33004, United States of America; 2 Marine Biological Association of the United Kingdom, The Laboratory, Citadel Hill, Plymouth PL1 2PB, UK; 3 Danah Divers, Marine Research Facility, PO Box 10646, Jeddah, 21443, Saudi Arabia; 4 University of Plymouth, Drake Circus, Plymouth, Devon, PL4 8AA, UK; 5 Department of Biological Sciences, University of Rhode Island, Kingston, Rhode Island, United States of America; 6 CIBIO – Universidade do Porto, Centro de Investigação em Biodiversidade e Recursos Genéticos, Campus Agrário de Vairão, Rua Padre Armando Quintas, 4485-668 Vairão, Portugal; 7 Bermuda Shark Project, Bermuda; 8 Ocean and Earth Science, National Oceanography Centre Southampton, University of Southampton, Waterfront Campus, European Way, Southampton SO14 3ZH, UK; 9 Instituto de Investigaciones Marinas, CSIC, Eduardo Cabello 6, 36208, Vigo, Spain; 10 CETMAR, Eduardo Cabello 6, 36208, Vigo, Spain; 11 Centre for Biological Sciences, Building 85, University of Southampton, Highfield Campus, Southampton SO17 1BJ, UK

## Abstract

Long-distance movements of animals are an important driver of population spatial dynamics and determine the extent of overlap with area-focused human activities, such as fishing. Despite global concerns of declining shark populations, a major limitation in assessments of population trends or spatial management options is the lack of information on their long-term migratory behaviour. For a large marine predator, the tiger shark *Galeocerdo cuvier*, we show from individuals satellite-tracked for multiple years (up to 1101 days) that adult males undertake annually repeated, round-trip migrations of over 7,500 km in the northwest Atlantic. Notably, these migrations occurred between the highly disparate ecosystems of Caribbean coral reef regions in winter and high latitude oceanic areas in summer, with strong, repeated philopatry to specific overwintering insular habitat. Partial migration also occurred, with smaller, immature individuals displaying reduced migration propensity. Foraging may be a putative motivation for these oceanic migrations, with summer behaviour showing higher path tortuosity at the oceanic range extremes. The predictable migratory patterns and use of highly divergent ecosystems shown by male tiger sharks appear broadly similar to migrations seen in birds, reptiles and mammals, and highlight opportunities for dynamic spatial management and conservation measures of highly mobile sharks.

Migration is typically identified as persistent, straightened movement that requires temporary inhibition of station-keeping behaviour, and is recognised as an adaptation driven by the transitory availability and location of resources^1^. In this context, migration is ubiquitous across animal taxa and its elucidation has been an important component in a wider understanding of animal population ecology^1^. Generally, this is because temporal change in the density of a population at a specific geographic location is not only a function of births and deaths but also of movements, including migration^2^. However, long-term tracking studies have focused largely on terrestrial and aerial species, with the most commonly identified (‘classical’) form of migration involving seasonal movements between a breeding and non-breeding area^1^. The availability of remote marine telemetry systems in recent years has enabled increasing studies tracking marine predators, such as turtles, seabirds and marine mammals, many of which reveal long-distance movements consistent with population-level migration^3^,^4^,^5^. By comparison, a general understanding of migratory behaviour in large sharks is less well developed, in part due to still few studies achieving multi-year tracks to detect repeated seasonal patterns^4^,^6^,^7^,^8^,^9^,^10^,^11^. Determining the timing, repeatability and potential motivations for annual movements of large sharks is necessary to understand the ecological and evolutionary role of such behaviour more generally in marine predators.

Global exploitation of large pelagic fish by industrialised fisheries has resulted in dwindling catches of important stocks despite increasing fishing effort^12^, emphasising the urgent need for enhanced management and conservation efforts^13^. Management action ideally necessitates evidence of population-wide declines but there is controversy^14^,^15^ over whether reported declines in shark catch rates within analysed regions reflect decreasing population abundance over entire ranges^16^,^17^, or are confounded by shifts in shark movements and habitat selection and changes in the areas exploited by fisheries^18^. More reliable interpretation of population size trends from shark fishery catch data will benefit from identifying the migratory ranges, routes and residency patterns of exploited species, particularly in the Atlantic where there is little appreciation of the spatial dynamics of overlap between sharks and fishing fleets despite fishing exploitation being exceptionally high^19^,^20^. With few exceptions^4^,^6^,^7^,^9^,^10^, detailed, long-term movement information remains sparse for many large shark species, making it very difficult to assess the potential efficacy of oceanic Marine Protected Areas (MPAs) for these highly mobile species^21^.

The tiger shark *Galeocerdo cuvier* (Péron & Lesueur, 1822) is an interesting and suitable species to investigate migratory patterns because it is one of the largest predatory sharks, reaching up to *~*5.5 m in length and ~600 kg in mass, and is found circumglobally in tropical and warm temperate coastal/pelagic waters^22^. It is captured in commercial fisheries, and is listed as ‘near threatened’ in the Red List of the International Union for Conservation of Nature (IUCN)^23^. The tiger shark typically occupies the highest trophic level available where it occurs, often being the sole predator on a wide range of other large, highly mobile marine vertebrates (e.g. marine mammals, turtles, other elasmobranchs)^24^,^25^,^26^,^27^. Moreover, tiger sharks have a very cosmopolitan diet and, consequently, are highly connected in marine food webs, displaying a wide niche breadth that is mostly attributable to high individual variation in prey consumed and depth utilisation^26^,^28^. A wide niche breadth of a predator could indicate an adaptation allowing it to remain within relatively localised areas, thus foregoing the necessity for seasonal migration to specific foraging grounds to feed on seasonally abundant prey. But several studies have documented long-distance movements for individual tiger sharks^8^,^28^,^29^,^30^,^31^,^32^,^33^,^34^. Additionally, seasonal variation in movement behaviour has been inferred from non-continuously tracked animals in acoustic telemetry-based presence/absence studies^8^,^35^. However, detailed spatial behaviour observed by continuous tracking over multiple years consistent with more classical, seasonal migratory patterns between discrete focal habitats has not been described.

In this study we use long-term satellite tracking of tiger sharks to determine movement patterns across multiple years, including examination of whether a large, marine predator with high intraspecific variability in diet and vertical habitat use shows any predictable migratory behaviour.

## Results

We tagged a total of 24 tiger sharks, 20 of which were male, varying in total length (TL) from 1.73 to 3.96 m (mean 3.03 m; Supporting Information, table S1). Overall, tiger shark movements were tracked for a total of 411 months (mean 17.1 months), covering an estimated distance of 356,085 m (mean 14,836 km), averaging 865.3 km month^−1^. Tracking periods for individual sharks ranged from 41 to 1101 days (mean 514 d), generating between 19 and 2,404 geolocations (mean 821) of varied Argos location class. Four individuals experienced intermediate transmission absences of 100 days or more. None of the sharks showed evidence from their SPOT transmissions of being captured during their tracks (e.g. a sudden sequence of LC3s).

### Repeated, long-distance migration

**Figure 1:**
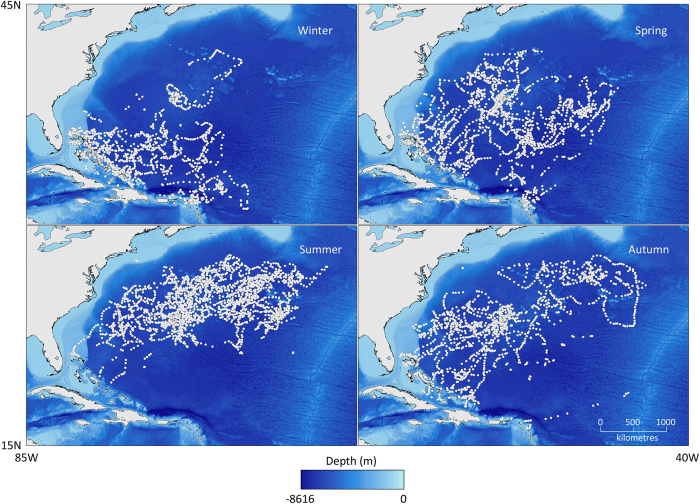
SSM adjusted geolocations for all tiger sharks separated by season and overlaid on bathymetry. Maps created in ArcGIS, using GSHHG coastline data and ETOPO2v2 bathymetry data.

Tiger sharks tagged at Bermuda displayed extensive space-use throughout the northwest Atlantic, ranging between latitudes of 17–40° N and longitudes of 48–79° W (Fig. 1), covering 6.7 million km^2^, as determined by the 95% isopleth of a kernel density plot for all sharks. This space-use varied seasonally, however, revealing long-distance north-south migrations (Fig. 1). Locations occupied during winter were primarily associated with coral reef-bound islands in the Bahamas, Turks and Caicos Islands, and Anguilla/Saint Martin. None of the tiger sharks was recorded entering the Caribbean Sea, nor crossing the mid-Atlantic Ridge. In contrast, during summer the majority of sharks adopted a temperate, oceanic habit, with most occupying open water north/northeast of Bermuda. There was a more dispersed distribution of locations in both spring (sharks generally moving north) and autumn (generally moving south), representing migratory transitions between the winter insular and summer oceanic phases.

### Partial migration

**Figure 2:**
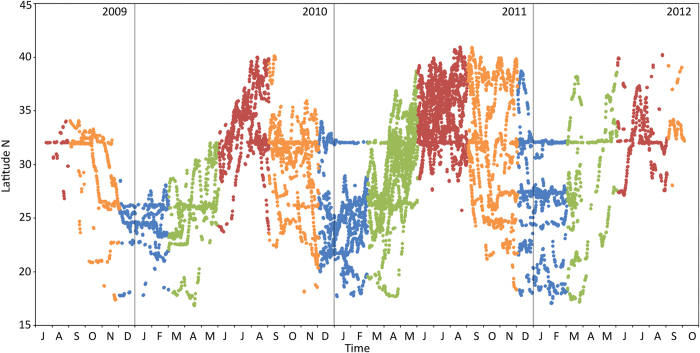
Latitude of all tiger shark locations over time (2009–2012), colour coded by season (blue = winter; green = spring; red = summer; orange = autumn).

The majority of tiger sharks (16; 273–396 cm TL) displayed a seasonal pattern of considerable latitudinal displacement (up to 2,500 km), between southern islands in winter and northern oceanic areas in summer (Fig. 2). The precise timing and duration of these migrations varied both between years and individuals. Notably, the five smallest tagged sharks (two females and three males: sharks 5, 12, 13, 15, and 20; 173–259 cm TL; table S1) did not conform to this general seasonal migratory pattern, staying in the vicinity of Bermuda over winter (Figs. 1,2). The two largest of these Bermuda overwintering residents (12 and 13, both 259 cm TL at tagging) did eventually undertake longer distance movements, but not until eight and eleven months after tagging, respectively, and neither migrated in the first winter season of their tracks. Overall, larger sharks tended to travel at increased rates (Spearman rank correlation between mean number of kilometres travelled per month and shark total length: *r*_s_ = 0.58, p < 0.01). Although only four female sharks were tracked, both patterns – seasonal migrations and Bermuda winter residence – were displayed by both sexes.

**Figure 3:**
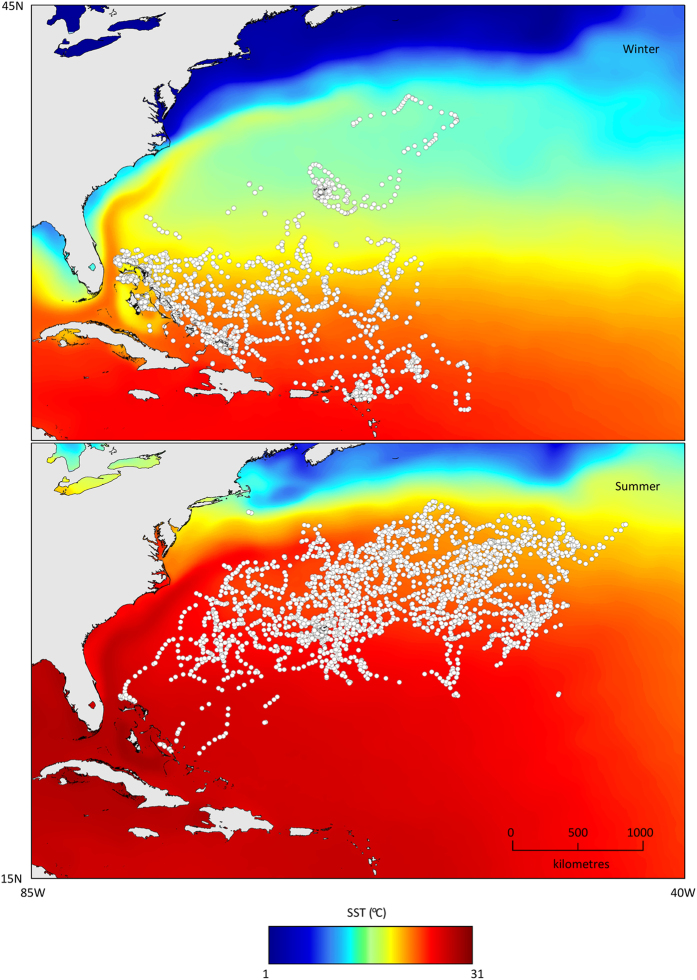
SSM corrected geolocations for all tiger sharks in winter and summer, overlaid on mean seasonal sea surface temperature (SST). Maps created in ArcGIS, using GSHHG coastline data and OSTIA SST data.

During winter, migratory individuals occupied the warmer, southern waters of the northwest Atlantic, and the expansion in range north during the summer coincides with warmer waters (>25 °C) extending up to the Gulf Stream (Fig. 3). The mean sea surface temperature (SST) of the southern insular regions exceeds that of the northern oceanic area throughout the year; however only during late summer and early autumn (July, August, September) does the mean SST in the north exceed the mean winter SST in the southern extent (Supporting Information, Fig. S3). Consequently, the individuals that undertook the annual north-south migrations occupied waters with surface temperatures of approximately 24–26 °C in both winter and summer, whereas those remaining near Bermuda over winter experienced lower surface temperatures (18–20 °C).

Despite the large range of movements by most tiger sharks, high occupancy was spatially restricted while in insular southern areas: up to 6–12 weeks within a given 0.5° × 0.5° cell (Supporting Information, Fig. S4a). In contrast, occupancy in oceanic areas was considerably more transient: little time was spent in any given oceanic cell, although there was elevated space-use around Bermuda, especially Challenger Bank, in the northeast of their tracked range.

### Philopatry

**Figure 4:**
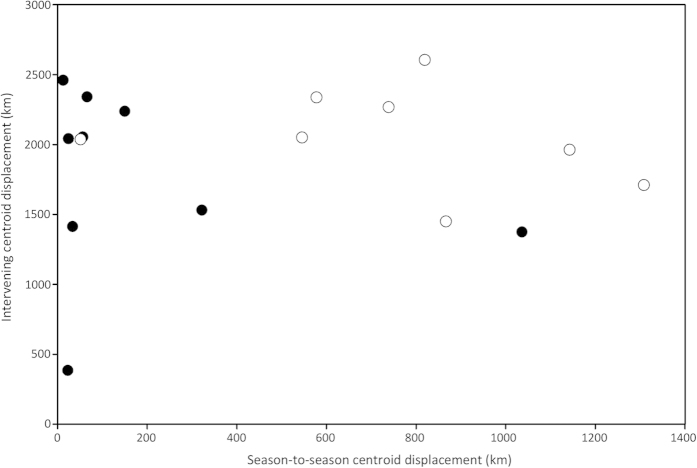
The relation between season-to-season centroid displacement (‘•’ = winter; ‘○’ summer) and the intervening centroid displacement for both successive winters and summers, from sharks with tracks of two years or more.

**Figure 5:**
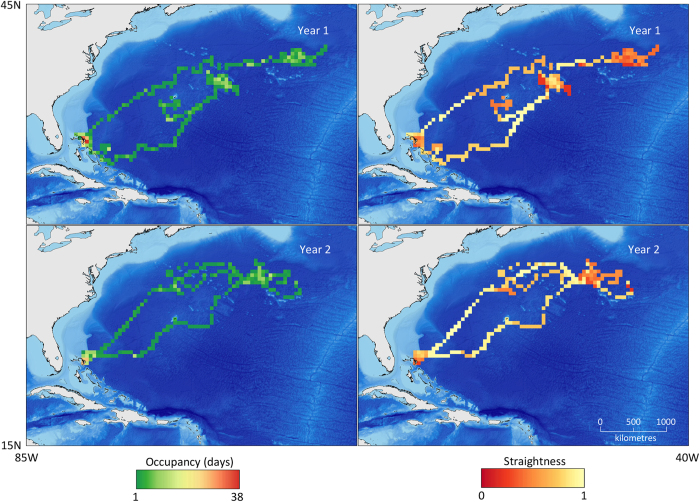
The occupancy and mean straightness of movement for shark 7 (384 cm male) for the first and second year of its track (measured from tagging date). Maps created in ArcGIS, using GSHHG coastline data and ETOPO2v2 bathymetry data.

There were nine individuals with enough data to investigate seasonal migratory philopatry across two or more years, six of which displayed distinct repeatability in the locality of their space-use. Winter philopatry was high, whilst summer philopatry appeared low (Fig. 4). The mean winter-to-winter centroid displacement was 191.4 km (ranging 12.4–1036.2 km, SD 331.6 km), whereas the mean summer-to-summer centroid displacement was 756.1 km (ranging 51.0–1308.2 km, SD 386.2 km). The repeated, philopatric migration pattern is exemplified by shark 7, which displayed spatially restricted use of a particular insular region and offshore oceanic regions over 3,500 km away, punctuated by relatively direct dispersals (Fig. 5). In both years of its two year track, shark 7 occupied the same area in the Bahamas during winter, displaying a winter-to-winter centroid displacement of only 65.7 km, although its centroid displacement between summers was 819.2 km. Over a three year track, shark 1 displayed similar insular winter philopatry (centroid displacements of 24.3 and 56.2 km), but also some degree of philopatry to offshore areas over 2,500 km away across consecutive summers, with summer-to-summer centroid displacements of 51.0 km and 545.3 km. In contrast, use of insular areas by shark 4 was comparatively dispersed, spending no more than 13 days within any given cell and providing multiple centroids for each season (Supporting Information, Fig. S5).

### Straightness of movement

Analysis of the comparative straightness of shark movements revealed overall reduced straightness around the southern islands, and also on the northern edge of the recorded range adjacent to the Gulf Stream. In contrast, shark movements were more directed in the oceanic environment in between these locations (Supporting Information, Fig. S4b). Despite lower occupancy compared to insular regions, the north-eastern area of the tracked sharks’ range (south of the Flemish Cap and in the general proximity of the Corner Rise Seamounts) is an area of particularly high turning frequency. Considering only summer straightness of movement emphasises this high turning frequency further (Supporting Information, Fig. S6). Overlaid with the juvenile loggerhead turtle *Caretta caretta* tracks of McClelland and Read (2007) and Mansfield *et al.* (2009), this area of high tiger shark turning overlaps with the pelagic distribution of *C. caretta* both in summer and year round (Supporting Information, Fig. S6). These turtle tracks overlapped with 37.6% of the 0.5° × 0.5° cells in which the tiger sharks were recorded during summer. Moreover, the stomachs of four out of five tiger sharks opportunistically sampled from a commercial long-lining vessel contained *C. caretta*, including small juveniles consumed whole (Supporting Information, table 2; Fig. S6).

## Discussion

Our study is one of only a handful in obtaining multi-year, continuous, high resolution tracks of individual fish migrations^4^,^6^,^7^,^8^,^10^,^11^, and provides the first report of annually repeated, distinct seasonal migrations for tiger sharks in the Atlantic. The satellite tracks are also the longest reported for individual tiger shark movements to date throughout their distribution (up to 1101 days, previously 517 days^34^). This apex marine predator displays remarkable plasticity in ecosystem use, accomplished by extensive, seasonal migrations between insular, coral reef ecosystems in winter and temperate oceanic, potentially foraging areas in summer. These round-trip migrations span over 7,500 km annually, with individuals displaying marked philopatry to overwintering areas. These migrations are also partial in nature: the five sharks that remained close to Bermuda over winter were all juveniles (including both sexes), whilst all migrants were large males, with the exception of the single mature female tracked.

Use of disparate, contrasting habitats is common among diadromous fish, but the repeated switching between such markedly different ecosystems (in terms of thermal regime, bathymetry, structural complexity and insular coral reef to oceanic ecosystems) as we show here for the tiger shark is not commonly reported for marine fish species. Consequently it is particularly notable that the sharks we tracked invested in dual strategies, switching between highly focused use of insular reef systems and dynamic use of open ocean, in addition to exhibiting strong, repeated migratory philopatry to overwintering sites. Philopatry may improve foraging success and be less costly than searching for other suitable habitat elsewhere, potentially enhancing individual fitness^36^.

Few marine fish have been shown to adopt such marked behavioural plasticity in ecosystem use, in particular repeated within individuals across years. The closest parallel reported among elasmobranchs is for endothermic sharks in contrast to the ectothermic tiger shark. For example, the white shark *Carcharodon carcharias* in the Pacific and Indian Oceans switches between high fidelity to particular coastal areas and long-distance migrations to oceanic areas^7^,^9^,^37^. The closely related salmon shark *Lamna ditropis* also makes long-distance migrations offshore in the Pacific Ocean, before returning to specific regions of the Alaskan coast^6^. For ectothermic sharks, philopatry to tropical insular regions has been shown for the sympatric oceanic whitetip shark *Carcharhinus longimanus*, which returns to particular areas of the Bahamas after movements into the Atlantic^38^, however this behaviour has not been demonstrated across multiple years nor across as vast oceanic distances as displayed by the tiger sharks. Among teleosts, some large, temperate, demersal species such as Atlantic cod *Gadus morhua* are known to return to within a few kilometres of the previous year’s spawning sites, despite long-distance migrations in between to foraging grounds^39^. However, the behaviours displayed by the tiger sharks migrating between tropical islands and distant, higher latitude, temperate oceanic zones are seemingly more similar to some turtle, bird and mammal movements than to other fish. For instance, loggerhead turtles display a marked dichotomy of ranging behaviours, switching between coastal and oceanic habits, often returning to within a few kilometres of previous foraging sites^36^,^40^. Leatherback turtles *Dermochelys coriacea* display similar seasonal movements, associating with aggregations of gelatinous zooplankton in the Irish Sea in summer^41^. Among birds, Cory’s shearwaters *Calonectris diomedea* in the Atlantic undertake long-distance, trans-equatorial, round-trip migrations between particular nesting sites and foraging areas^3^, as do sooty shearwaters *Puffinus griseus* in the Pacific^42^. Baleen whales, such as the humpback whale, *Megaptera novaeangliae*, exemplify similarly substantial repeat migrations in mammals, which move thousands of kilometres seasonally between near-polar feeding grounds and tropical breeding grounds^43^. Southern elephant seals *Mirounga leonina* have also been demonstrated to show very high fidelity to offshore foraging areas in the Antarctic between years^44^.

Understanding the motivations behind such migrations will better enable prediction of how movements might respond to environmental changes. However, despite a number of tracking studies correlating animal movements with environmental variables^4^,^8^,^45^,^46^, the motivation for migration often remains unknown^7^,^8^,^32^. The tracked tiger sharks migrated north in spring and summer as sea surface temperatures increase, displaying very high turning frequencies in the north and north eastern extent of their range, which may reflect potential foraging activity^47^. Another ocean migrant, the leatherback turtle, displays similarly high foraging activity at higher latitudes, following extended migration from tropical waters^41^. In addition, the northerly limit of tiger shark movements may be driven by thermal preferences, as it appears from comparisons with seasonal SST that their movements are contained within an isotherm of approximately 24 °C. Isotherms are thought to drive range limits of other ectothermic species, such as leatherback turtles, which also undertakes large north-south movements in the Atlantic^48^. Consequently a conceivable motivation for the sharks to migrate in the summer may be foraging opportunities in the area, including on juvenile turtles, cued by increasing sea surface temperature. Elsewhere turtles make up a significant portion of the diets of larger individual tiger sharks^24^,^25^, so it is possible that the tracked tiger sharks may migrate to exploit an abundance of preferred prey in the summer, connecting the trophic ecologies of disparate coral reef and oceanic ecosystems. However, this hypothesis remains untested and requires further investigation; for instance turtles may simply appear more prevalent in a diet if their shells digest more slowly than other items.

As the majority of sharks tagged in our study were mature males, a possible reason for them to return from foraging to their overwintering areas is to find mates. Consistent with our study, some large female tiger sharks tracked from the Bahamas have also travelled long distances into the Sargasso Sea, but most remained relatively close to the Bahamas and Florida^29^, where there is an apparent peak in pupping during early summer^49^. Given that tiger sharks in the northwest Atlantic have a 13–16 month gestation period^50^, mating should have peaked in late winter/early spring, when adults of both sexes are known to be in tropical insular regions. Although other factors may be involved, including foraging and thermal preferences, given the available information it is reasonable to hypothesise that a driver of winter philopatry is returning for mating opportunities.

Complex population structure and extensive movements by a segment of the population can result in regional fishing activity having disproportionate effects on different population components^19^. Thus, understanding potential demographic segregation and partial migration patterns – who goes where, when and why – is crucial for the sustainable management of any population. Partial migration is widespread across taxa, although the driving processes often remain unclear, with animal size, sex, condition and personality (e.g. boldness) all reported as factors contributing towards the propensity to migrate or not^51^. Partial migration has been reported for female tiger sharks in Hawaii based on presence/absence data from acoustic telemetry, where seasonal presence appears to be associated with reproductive state and individual foraging targets^8^. From work on other species it has been suggested that swim speed and migration propensity and ability may be linked to size-related dispersal ability^51^,^52^. This is consistent with the observation in the present study that distance travelled per month increased with tiger shark length and, furthermore, that all individuals observed overwintering around Bermuda were comparatively small and immature^50^. Similarly in Hawaii larger tiger sharks were also more likely to undertake long range movements^8^, and year-round residency has been reported for sub-adult tiger sharks at the Chesterfield Islands in the Coral Sea^32^. Work on salmonids *Coregonus* spp. suggests that smaller individual fish within an ectothermic species may incur a greater metabolic cost in warmer waters, potentially reducing the benefits of migration^53^. If such a size-dependent limitation on long-distance dispersal were applicable to tiger sharks, it would be consistent with our observation of fewer smaller individuals migrating seasonally to exploit prey elsewhere and remaining within cooler water over winter. The overwintering of smaller, immature sharks in cooler waters is also consistent with the hypothesis of mating as a driver for southerly migrations of mature individuals.

Individual condition may therefore be a strong driver of migration propensity in tiger sharks: adults may be of sufficient condition to absorb the costs of migration to exploit disparate, but profitable, food sources, with females possibly skipping migration if gravid, whilst juveniles may have to invest more in somatic growth.

Such segregated use of large oceanic areas by size, as shown here, combined with high fidelity to particular regions, can result in differential exploitation by spatially-focused fisheries and contribute towards rapid population declines^19^,^54^. With the observed size-related migration differences in tiger sharks, such differential exploitation by long-line fisheries in summer could disrupt the age structure of the population, exacerbating any impact of fisheries-induced mortalities. Some overwintering sites are covered by the Bahamian Exclusive Economic Zone, where long-lining and commercial trade of shark is prohibited, whereas sharks migrating to oceanic areas may be at greater risk of fishing mortality. This highlights the need for informed, spatially dynamic, management and conservation measures, such as the designation of MPAs or time/area closures of fisheries in summer foraging areas, or for greater spatial protection of philopatric overwintering sites.

Our study reveals unexpected predictability in tiger shark horizontal movements in the northwest Atlantic, which contrasts with the high intraspecific variability observed in their vertical movement behaviour in the same region^28^. They seasonally and repeatedly switch between coastal coral reef and temperate oceanic habitats, displacing thousands of kilometres in the process, yet also showing marked philopatry to overwintering sites. However, the expansive movements of tiger sharks throughout the northwest Atlantic leaves them exposed to international fisheries for extended periods of time. Understanding these migration patterns, particularly when partial in nature and size segregated, is crucial for future conservation efforts. Identifying where tiger sharks may focus their movements and use migration corridors will inform assessments of where, when and how high space-use areas overlap with commercial fisheries in the North Atlantic.

## Methods

We tagged 24 tiger sharks with Argos satellite platform terminal transmitters, or PTTs (SPOT5, Wildlife Computers, Redmond, Washington, USA) between August 2009 and July 2012 at Challenger Bank (N 32°05’, W 065°03’) near Bermuda in the northwest Atlantic (Supporting Information, table S1). All field work was approved by, and conducted with the knowledge of, the Marine Resources Section of the Bermuda Department of Environmental Protection. The shark handling and tagging methods were performed in accordance with the approved guidelines of Nova Southeastern University. The SPOT5 tag location accuracy is determined by the timing and number of transmissions received by Argos satellites within a single overpass^55^. The location classes (LCs) available are 3, 2, 1, 0, A and B, with LC3 providing the lowest errors and LCB the highest^56^,^57^.

As Argos positions vary in frequency and quality it was necessary to process the data to obtain normalised positions that were comparable between individuals and over time. The raw Argos positions were processed in three steps, each adopted to address a specific issue. Firstly, it was necessary to avoid inclusion of steps between positions that were deemed too large to be biologically plausible, basing filter rules on previously documented swimming speeds for large sharks^58^. To do this we analysed all raw positions point-to-point with a 3 m s^-1^ swim speed filter and 20 km distance filter: any position separated from both adjacent positions by either too great a distance or speed were shifted to a linearly interpolated position between the two (i.e. the most parsimonious location). Positions where either the distance or speed to only one of the adjacent positions was too great were ignored. Secondly, because each raw position has a different error field according to its Argos location class, we needed to decide the most probable location for each point within its error field. We achieved this by using a Bayesian state-space model (SSM) that adjusted the filtered tracks by producing regular positions based on the Argos location class, mean turning angle, and autocorrelation in speed and direction, producing the most probable track through the error fields^59^. Given that 80.1% of gaps between positions in our tracks were under 12 hours (Supporting Information, Fig. S1), we used a time step of 12 hours in the SSM to produce two positions per day for each shark’s track. However, the SSM produces regular positions for the entire track, even on days where there were no raw positions. Consequently we deleted all positions for days on which there were no real Argos transmissions. This step resulted in our normalised track positions and formed the dataset used for the plotting of positions on maps by season and plotting latitude over time to display how the distribution of animals changes over time.

Argos tracks only have locations for when the sharks were at the surface; consequently there is high variability in the number of locations in a given area, as a result of the shark’s varied surfacing behaviour rather than because of its actual location. This would introduce a bias into the analysis of time spent in different areas. To correct this bias, linear interpolation was used to normalise the transmission frequency by generating points at 12 hour intervals along track gaps of <20 days. Where gaps >20 days were encountered the track was split into sections to avoid spurious interpolation. Moreover, in order for these space-use analyses to be as conservative as possible, all were conducted at a grid resolution of 0.5° × 0.5°, greater than the reported errors of the worst location class (LCB, ~10 km^56^,^57^). Examples of how track positions varied between each processing step can be found in Figure S2 of the Supporting Information.

To determine track sections with higher turning frequency from those with more directed movement, the ‘straightness’ of individual trajectories was calculated for successive 12 day portions of each SSM processed, linearly interpolated track, where:

Straightness = displacement over 12 days / distance travelled over 12 days

Values closer to 1 indicate periods of straighter movement, and values closer to 0 indicate periods of higher turning frequency, providing a proxy for station-keeping or area-restricted searching (foraging) behaviour^47^. Straightness was calculated over 12 day periods as this was, on average, the time taken for the sharks to traverse a distance greater than the error of the worst location class (LCB, ~10 km^56^,^57^). The mean distance travelled per month was also calculated for each individual, and correlated with individual total length using a Spearman rank correlation.

To perform analyses on space-use and movement behaviour, the SSM normalised, linear interpolated tracks were plotted on a 0.5°×0.5° grid cell in ArcGIS (ESRI Inc., CA, USA). Coastline and bathymetry data were obtained from the U.S. Department of Commerce, National Oceanic and Atmospheric Administration (NOAA): coastlines from the Global Self-consistent, Hierarchical, High-resolution Geography Database (GSHHG) and bathymetry from the 2-minute Gridded Global Relief Data (ETOPO2v2). Computerised digital images and associated databases are available from the National Geophysical Data Center, NOAA, U.S. Department of Commerce, http://www.ngdc.noaa.gov/. Sea surface temperature (SST) data were obtained from the Operational Sea Surface Temperature and Sea Ice Analysis (OSTIA) system via the U.K. National Centre for Ocean Forecasting (Contains public sector information licensed under the Open Government Licence v3.0 http://www.nationalarchives.gov.uk/doc/ open-government-licence/version/3/). All maps were created using the Plate Carrée projection.

The total time spent within each cell (occupancy) was calculated by summing the number of 12-hourly points located within cells. The mean straightness for each 0.5° × 0.5° cell was calculated by averaging the straightness values associated with points located within them. This was performed for all sharks combined as well as individuals, and for both complete tracks and tracks separated by season to address any seasonality in distribution. The seasons were defined as follows: Winter, Dec–Feb; Spring, Mar–May; Summer, Jun–Aug; Autumn, Sep–Nov. When occupancy was calculated for all sharks combined, the results were corrected for tagging location by dividing the occupancy value for each 0.5° × 0.5° cell by the number of tags active in that cell. The overall geographical range of tracked sharks was calculated in ArcGIS using the 95% isopleth of the kernel density estimate for all locations.

For qualitative comparison of seasonal distribution of locations with sea surface temperature (SST), track locations were overlaid in ArcGIS on seasonal SST means throughout the northwest Atlantic. In addition, the mean monthly SST for 5° × 2° areas at the northern and southern extents of the tracked sharks’ range were calculated to examine the SSTs likely experienced by sharks at the surface when in those areas compared to the typical annual variation in SST. The bounding for the northern extent was 37–39 °N by 62–57 °W, and for the southern extent was 24–26 °N by 76–71 °W.

A number of sharks displayed focused space-use in both winter and summer, so potential philopatry was tested for in individuals with sufficiently long tracks to cover repeat seasons (*n* = 9 sharks). First, central locations were calculated for individuals for each winter and summer period, defined as the central point, or centroid, of the 5% isopleth of the kernel density estimate for that season, and calculated using Geospatial Modelling Environment^60^. Season-to-season centroid displacement was then plotted against intervening centroid displacement for both successive winters and summers to test the spatial resolution at which sharks returned to a particular location given the intervening long-distance migration.

One of the authors (GRM) was opportunistically able to retain the stomachs of the five tiger sharks caught by a Spanish commercial long-lining vessel operating in the northwest Atlantic in 2012 for contents analysis whilst acting as a scientific observer on-board. The stomachs appeared to predominantly contain juvenile loggerhead turtles *Caretta caretta* (Linnaeus, 1758), and so maps of spatial and temporal variation in the straightness index were compared to the locations of juvenile loggerhead turtles as determined by satellite tracks reported in McClellan and Read (2007) and Mansfield *et al.* (2009). The loggerhead tracks were digitised using ArcGIS, where they were projected to the correct spatial reference and had their features recreated manually. To quantify any spatial overlap, the percentage of 0.5° × 0.5° grid cells in which both tiger sharks and loggerhead turtles were tracked was calculated in ArcGIS.

All shark tracks used in the present study are available for viewing on the Nova Southeastern University website: http://www.nova.edu/ocean/ghri/tracking/. However, given the tiger shark is listed as ‘near threatened’ in the IUCN Red List, the raw, detailed location data are considered sensitive information. Consequently the raw tracks are not freely available at present so as not to encourage further fisheries interactions.

## Additional Information

**How to cite this article**: Lea, J. S. E. *et al.* Repeated, long-distance migrations by a philopatric predator targeting highly contrasting ecosystems. *Sci. Rep.* **5**, 11202; doi: 10.1038/srep11202 (2015).

## Supplementary Material

Supplementary Information
