# Supplementary material for: Repeated, long-distance migrations by a philopatric predator targeting highly contrasting ecosystems
Source: Sci Rep. 2015 Jun 9;5:11202. doi: 10.1038/srep11202 (PMC4460898; doi:10.1038/srep11202)

Supporting Information for:

Repeated, long-distance migrations by a philopatric predator targeting highly contrasting ecosystems

by

James S. E. Lea, Bradley M. Wetherbee, Nuno Queiroz, Neil Burnie, Choy Aming, Lara L. Sousa, Gonzalo R. Mucientes, Nicolas E. Humphries, Guy M. Harvey, David W. Sims, Mahmood S. Shivji

Figure S1: Frequency distribution of time between subsequent geolocations for all sharks.

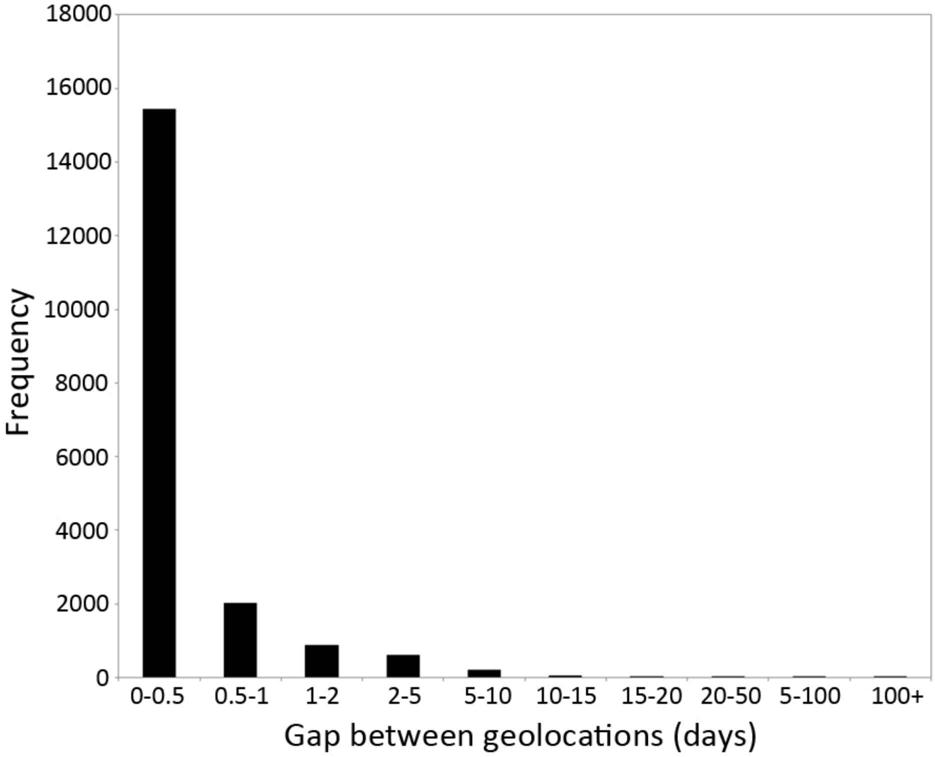

Figure S2: Maps to show how the positions varied between each stage of track processing for four different sharks (S7, large male; S12 small female; S15 small male; S16 large female): a = raw Argos positions, b = speed filtered positions, c = SSM positions, d = SSM positions with interpolation on data-less days, e = SSM positions with linear interpolation across gaps <20 days. Maps created in ArcGIS, using GSHHG coastline data and ETOPO2v2 bathymetry data.

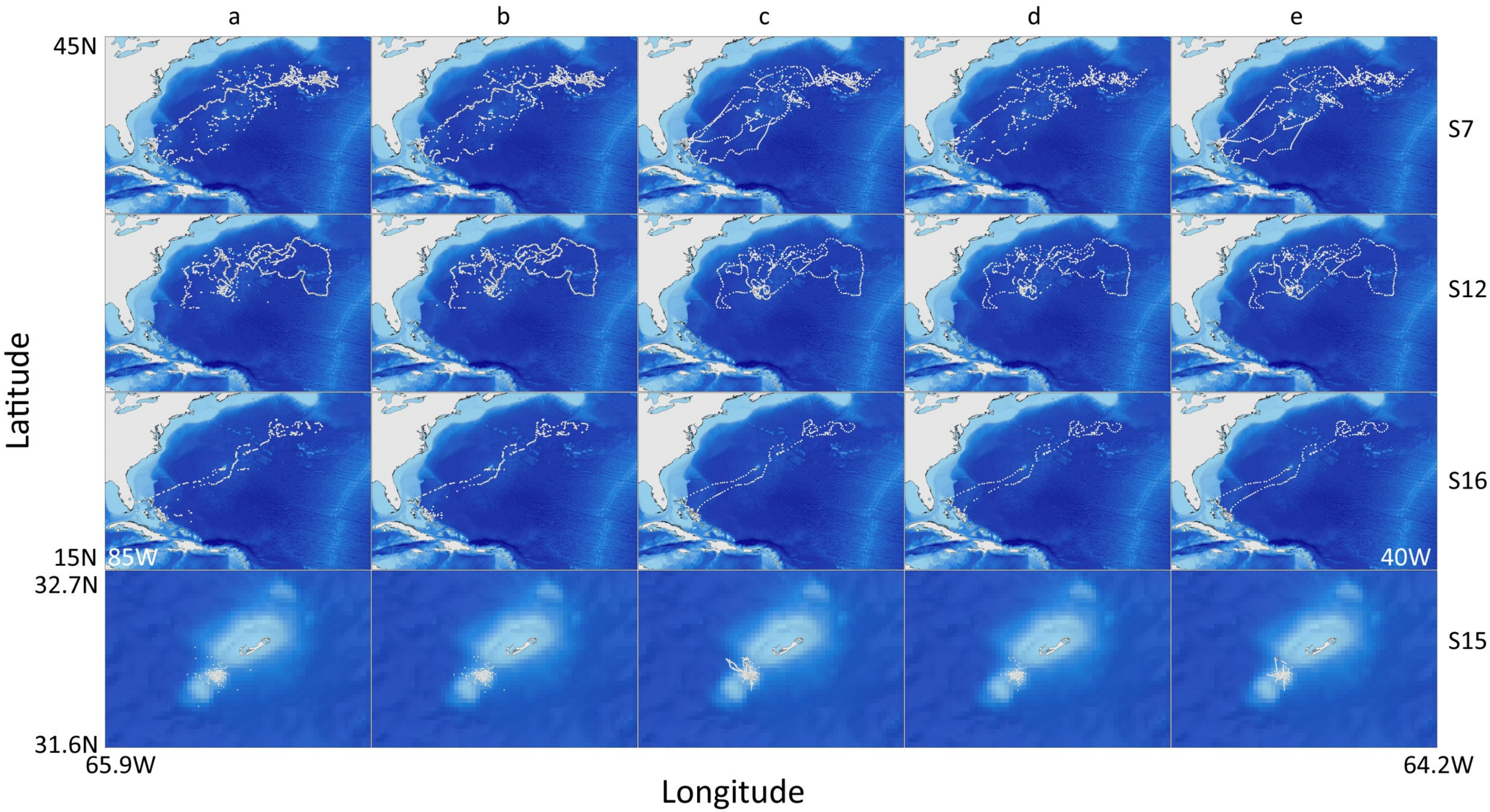

Table S1: Summary data for 24 tiger sharks tagged with SPOT5 transmitters at Challenger Bank, Bermuda.

ID	Sex	TL (cm)	Date tagged	Overall Detection Period (months)	Total Locations	Minimum distance (km)	Minimum distance/ month (km)
1	m	343	31-08-09	36.7	1163	42996	1172
2	m	334	19-07-09	32.6	83	9413	289
3	m	313	02-08-09	26.9	401	10540	392
4	m	361	29-07-10	25.8	1985	41158	1597
5	m	244	28-07-10	25.6	94	2431	95
6	m	295	03-08-09	24.9	1433	27723	1113
7	m	384	11-09-10	24.9	2404	31677	1274
8	m	371	07-09-10	24.8	809	26265	1058
9	m	333	28-07-10	24.6	1871	25066	1018
10	m	274	27-07-10	20.2	1628	19914	986
11	m	330	25-07-10	18.6	928	25012	1342
12	f	259	24-10-10	17.3	2352	19517	1128
13	m	259	14-10-10	14.0	335	7725	553
14	m	396	27-07-10	13.7	563	13081	953
15	m	216	18-08-10	13.4	285	622	46
16	f	354	16-08-09	13.1	1263	12197	933
17	m	346	05-08-09	13.0	312	15623	1199
18	m	292	25-07-09	12.2	279	5200	426
19	m	351	24-07-10	10.2	523	13083	1287
20	f	173	21-11-11	7.2	452	1292	179
21	m	305	28-07-10	5.9	19	1624	275
22	f	233	10-07-12	2.8	446	2224	804
23	m	348	28-07-10	1.9	38	1417	759
24	m	323	05-09-10	1.4	49	284	208

Figure S3: Mean monthly sea surface temperature (SST) for the northern and southern extents of the tracked sharks’ range. Figure created using OSTIA SST data.

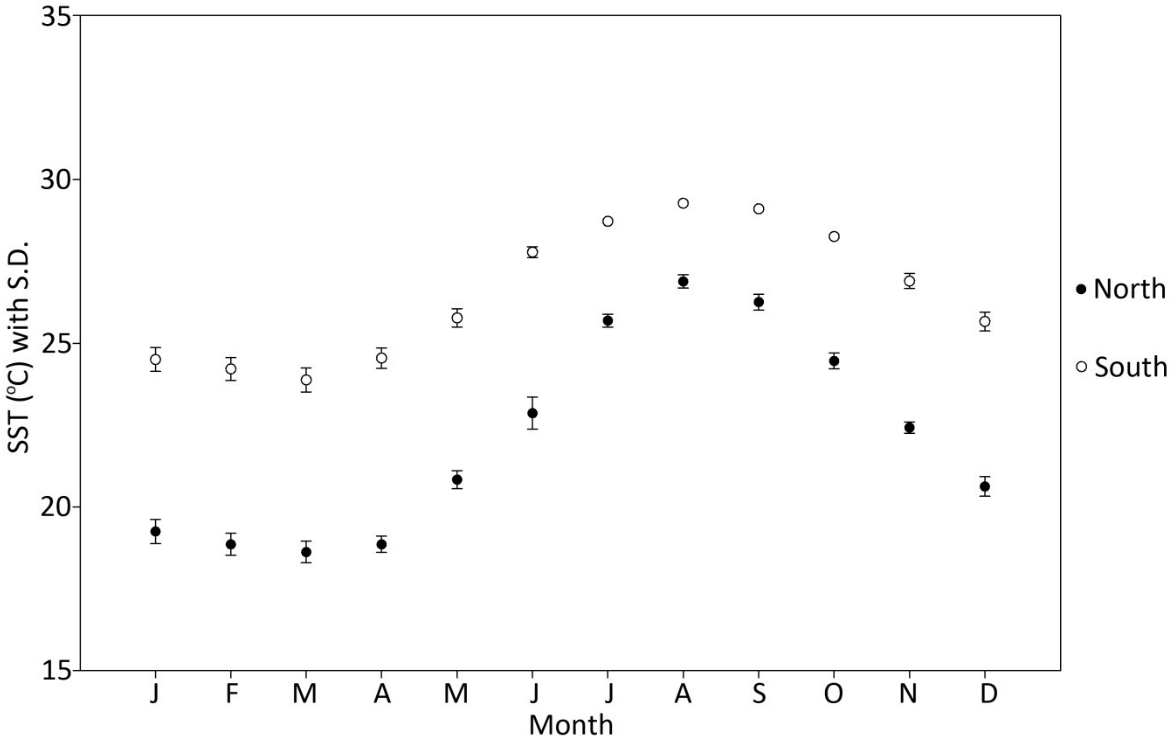

Figure S4: Maps of the overall occupancy (a) and mean straightness of movement (b) for all tiger sharks, overlaid on bathymetry. Maps created in ArcGIS, using GSHHG coastline data and ETOPO2v2 bathymetry data.

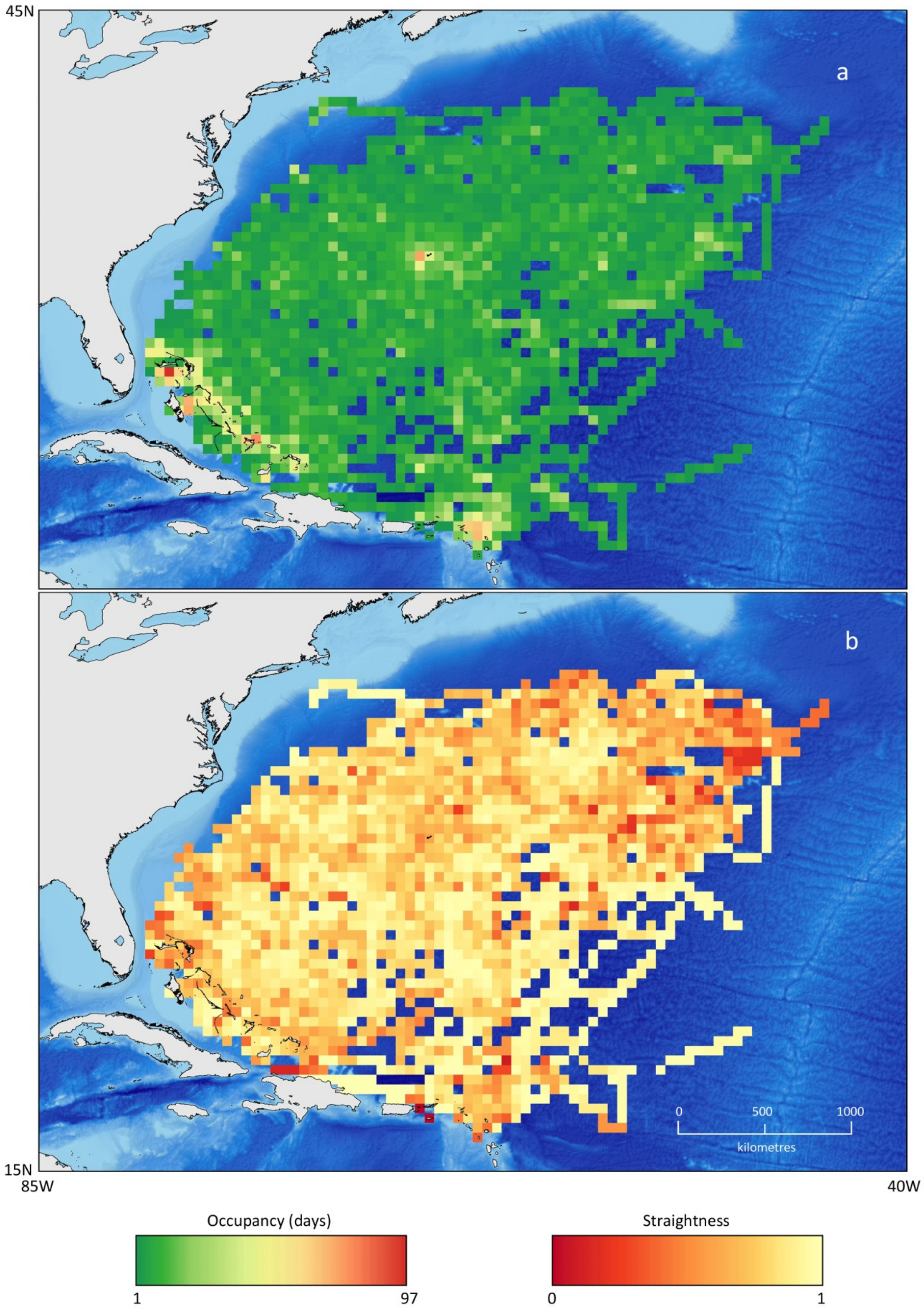

Figure S5: The occupancy and mean straightness of movement for shark 4 (361 cm male) for the first and second year of its track (measured from tagging date). Maps created in ArcGIS, using GSHHG coastline data and ETOPO2v2 bathymetry data.

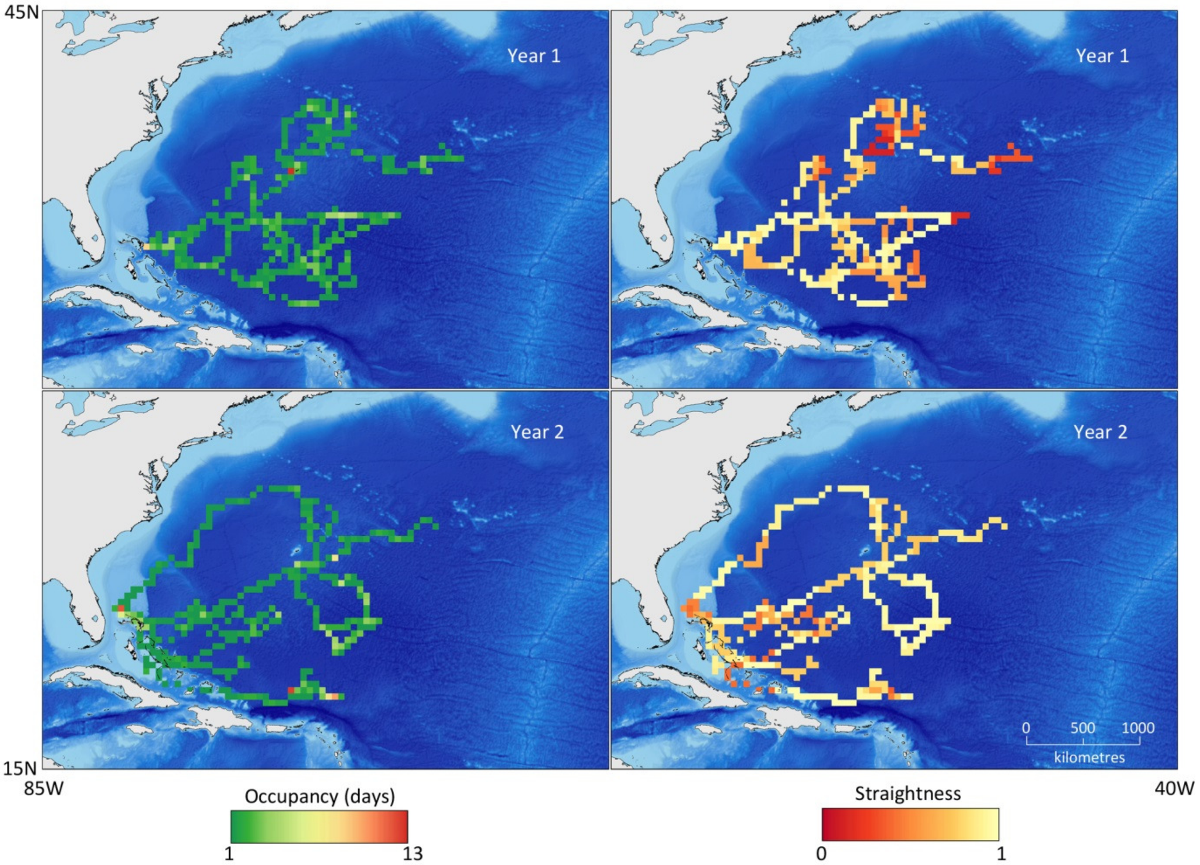

Table S2: Summary data of stomach contents from tiger sharks caught in the West Atlantic by a commercial long liner.

Shark	TL	Sex	Date	Lat	Long	Content (g)	*Caretta* %	Hydrobatidae %	*Balistes* %
T1	-	-	-	40	-49	140.8	100.0	-	-
T2	246	f	03-11-12	40.17	-49.11	0.0	-	-	-
T3	234	m	01-11-12	41.09	-48.14	200.8	66.1	0.5	33.9
T4	223	f	03-11-12	39.03	-49.37	92.0	98.9	1.1	-
T5	-	-	-	40	-49	156.8	100.0	-	-

Figure S6: Overall mean straightness of movement in summer, overlaid with juvenile *Caretta caretta* summer locations from Mansfield *et al.* (2009) and tracks from McClellan and Read (2007), as well as catch locations of tiger sharks from which stomach contents were obtained. Map created in ArcGIS, using GSHHG coastline data and ETOPO2v2 bathymetry data.

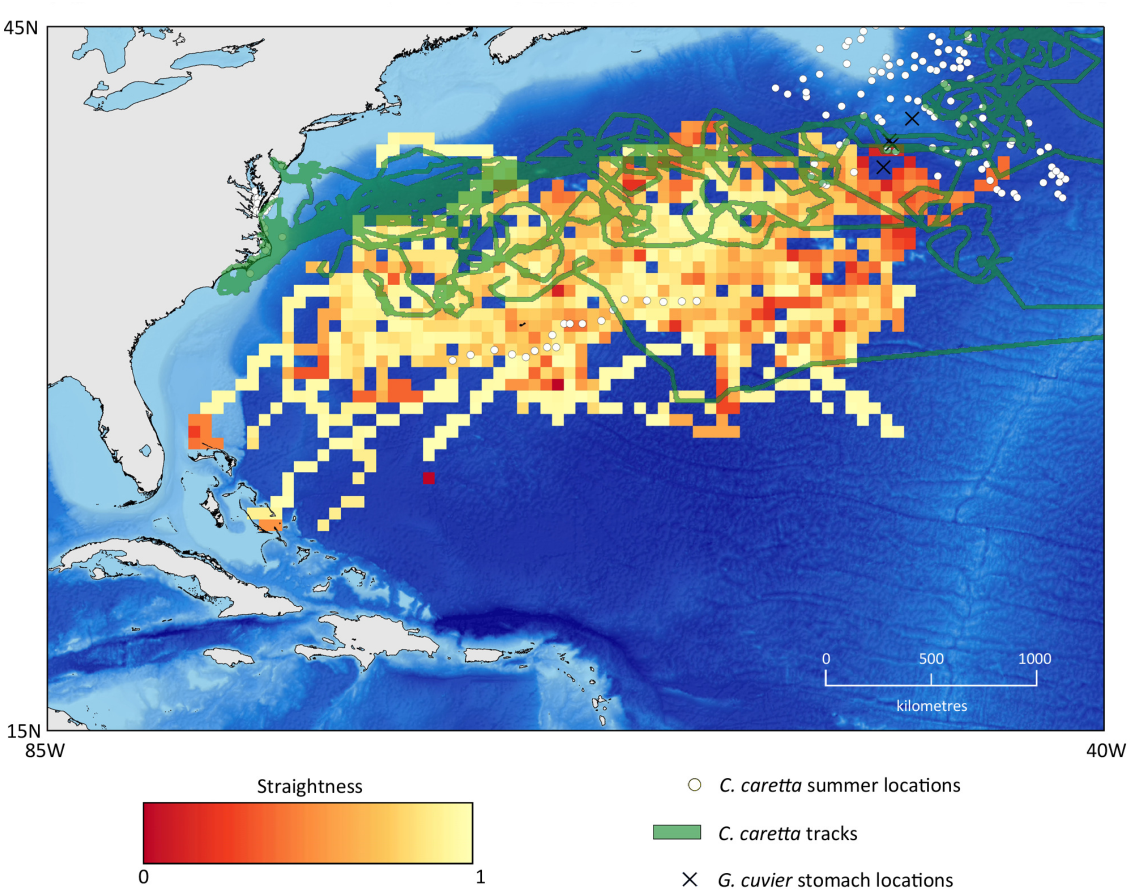

Supplement: Supplementary Information [file srep11202-s1.pdf]
